# Malaria incidence from 2005–2013 and its associations with meteorological factors in Guangdong, China

**DOI:** 10.1186/s12936-015-0630-6

**Published:** 2015-03-18

**Authors:** Cui Guo, Lin Yang, Chun-Quan Ou, Li Li, Yan Zhuang, Jun Yang, Ying-Xue Zhou, Jun Qian, Ping-Yan Chen, Qi-Yong Liu

**Affiliations:** State Key Laboratory of Organ Failure Research, Department of Biostatistics, School of Public Health and Tropical Medicine, Southern Medical University, Guangzhou, 510515 China; Department of Nursing, Faculty of Health and Social Sciences, The Hong Kong Polytechnic University, Hong Kong, China; State Key Laboratory for Infectious Disease Prevention and Control, National Institute for Communicable Disease Control and Prevention, Chinese Center for Disease Control and Prevention, Beijing, 102206 China; Department of Mathematics and Physics, School of Biomedical Engineering, Southern Medical University, Guangzhou, 510515 China

**Keywords:** Distributed lag non-linear model, Malaria incidence, Temperature, Duration of sunshine, Precipitation

## Abstract

**Background:**

The temporal variation of malaria incidence has been linked to meteorological factors in many studies, but key factors observed and corresponding effect estimates were not consistent. Furthermore, the potential effect modification by individual characteristics is not well documented. This study intends to examine the delayed effects of meteorological factors and the sub-population’s susceptibility in Guangdong, China.

**Methods:**

The Granger causality Wald test and Spearman correlation analysis were employed to select climatic variables influencing malaria. The distributed lag non-linear model (DLNM) was used to estimate the non-linear and delayed effects of weekly temperature, duration of sunshine, and precipitation on the weekly number of malaria cases after controlling for other confounders. Stratified analyses were conducted to identify the sub-population’s susceptibility to meteorological effects by malaria type, gender, and age group.

**Results:**

An incidence rate of 1.1 cases per 1,000,000 people was detected in Guangdong from 2005–2013. High temperature was associated with an observed increase in malaria incidence, with the effect lasting for four weeks and a maximum relative risk (RR) of 1.57 (95% confidence interval (CI): 1.06-2.33) by comparing 30°C to the median temperature. The effect of sunshine duration peaked at lag five and the maximum RR was 1.36 (95% CI: 1.08-1.72) by comparing 24 hours/week to 0 hours/week. A J-shaped relationship was found between malaria incidence and precipitation with a threshold of 150 mm/week. Over the threshold, precipitation increased malaria incidence after four weeks with the effect lasting for 15 weeks, and the maximum RR of 1.55 (95% CI: 1.18-2.03) occurring at lag eight by comparing 225 mm/week to 0 mm/week. *Plasmodium falciparum* was more sensitive to temperature and precipitation than *Plasmodium vivax*. Females had a higher susceptibility to the effects of sunshine and precipitation, and children and the elderly were more sensitive to the change of temperature, sunshine duration, and precipitation.

**Conclusion:**

Temperature, duration of sunshine and precipitation played important roles in malaria incidence with effects delayed and varied across lags. Climatic effects were distinct among sub-groups. This study provided helpful information for predicting malaria incidence and developing the future warning system.

**Electronic supplementary material:**

The online version of this article (doi:10.1186/s12936-015-0630-6) contains supplementary material, which is available to authorized users.

## Background

Malaria, one of the major global public health threats, has high morbidity and mortality despite relevant effective control in recent decades [1]. Globally, there were an estimated 207 million cases of malaria in 2012, and an increasing trend was observed in the annual numbers of malaria deaths [2,3]. It is well recognized that the prevalence and transmission of malaria are associated with multiple factors, such as meteorological, demographic and socio-economic factors, human behaviours that involve migration, excessive urbanization and air travel, and biological factors such as type of malaria vector and mosquito density [4-9]. Previous data have shown the apparent seasonal cycle of one year. The malaria incidence peaks in summer and autumn in most places, including China and Africa, and sometimes relatively small peaks can be seen during January and February [5,10,11]. This seasonal feature is dominantly driven by meteorological conditions, which affect mosquito abundance, feeding habits and life cycle period, and the parasite’s interaction with mosquitoes (e.g., the duration of sporogony) [6]. Some previous studies have shown that the intra-annual variation in malaria incidence may be associated with changes in ambient temperature, duration of sunshine, precipitation, wind speed, and relative humidity, etc., but there were inconsistent findings in the key factors observed and the corresponding effect estimates. For example, some studies reported significant effects of precipitation on malaria cases [4,6,10], while others found that the effects were governed by temperature and sunshine or no effects of precipitation were observed [7,12,13]. This discrepancy may be partly due to regional heterogeneity in climate and environment and different assumptions of various models (e.g., linear or non-linear effects assumed).

The climatic factors mainly affect the densities and activities of mosquitoes and the development of *Plasmodium* in mosquitoes, and there is an incubation period of seven days or longer between the bite of an infected mosquito and the onset of malaria symptoms [14]. Therefore, the effects of meteorological factors on malaria may be delayed. Many epidemiological studies assumed a linear relationship between climatic parameters and malaria [5,11], while it was not well documented whether there is a potential non-linear relationship. Most previous studies on environmental factors and malaria have adopted an autoregressive integrated moving average (ARIMA) model and its derived methods (SARIMA, GSARIMA and ARIMAX), which elucidated how climate could impact malaria and gave clues to prediction, but their applications were restricted by specific lags [15-18]. Several studies used regression analyses such as linear regression, logistic regression and a linear mixed model, which completely ignored the lagged effects of meteorological factors [5,19,20]. Recently, the distributed lag non-linear model (DLNM) has been applied in several studies [4,7,9,10], while uncertainties remain about potential effect modification by malaria type and individual characteristics, such as gender and age.

Malaria remains a serious health threat in China, particularly in southern China. The sub-tropical and tropical climate, accompanied by fast development of economic and population mobility, provide a favourable environment for *Plasmodium* and mosquitoes throughout the year, resulting in a potential threat of outbreak [4,10,11,21]. Guangdong, the largest and wealthiest province in southern China, suffered a lot from malaria in the past, but few epidemiologic studies of malaria have been conducted. This study aims to describe the epidemiologic features of malaria, investigate the non-linear lagged effects of climatic factors on malaria, and examine potential effect modification by individual characteristics in Guangdong, China.

## Methods

### Study site

**Figure 1:**
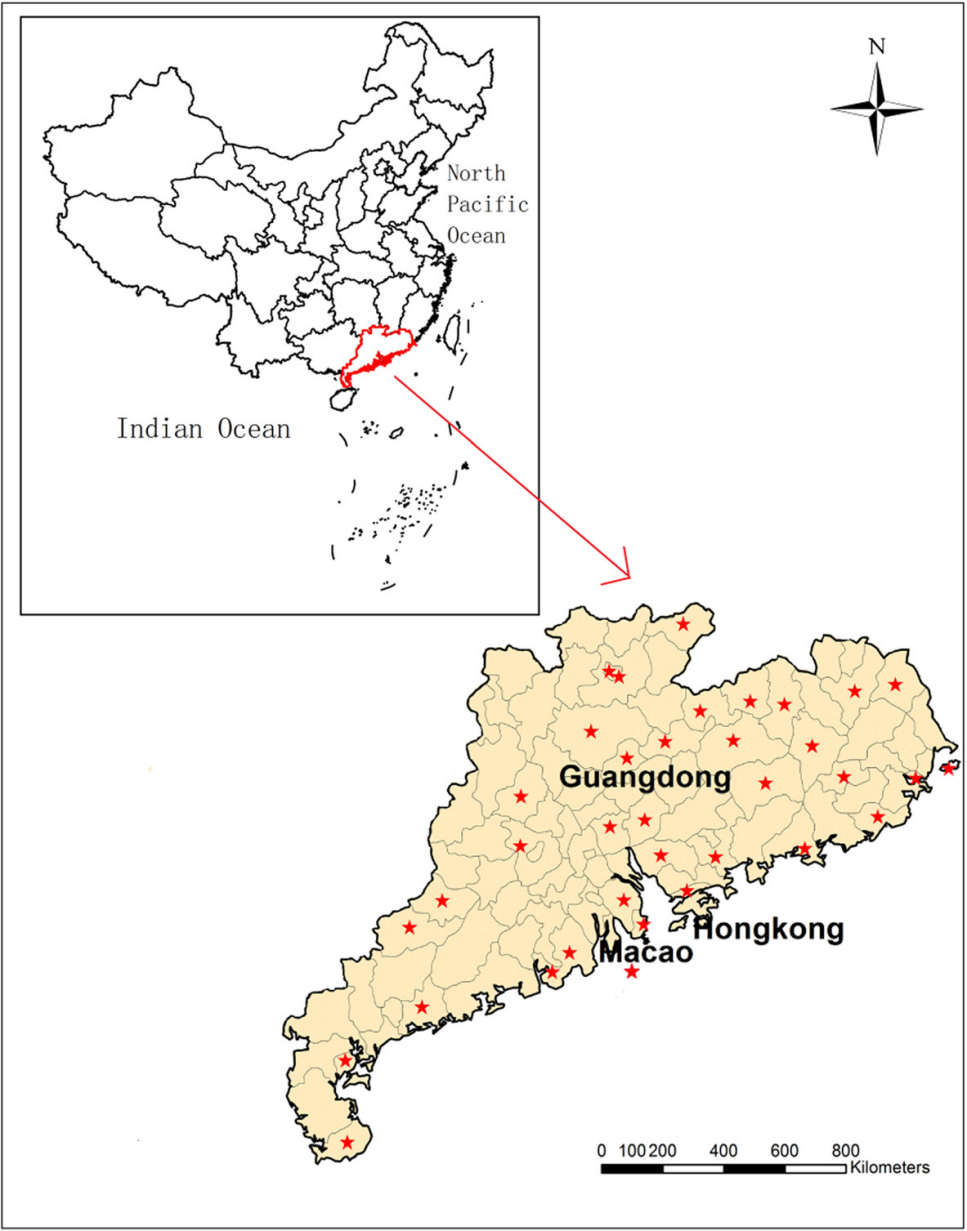
**Map of Guangdong.** The stars show the locations of weather stations in Guangdong.

Guangdong Province is situated at the southernmost tip of mainland China (20°18’ to 25°28’ N, 107°25’ to 109°45’ E) adjacent to Hong Kong (Figure 1). Guangdong has a population of 106 million residing in a land area of 179,612 sq km and a sub-tropical, marine, monsoon climate with an annual average temperature of 22°C and annual average rainfall of 1,500 mm.

### Data collection

In mainland China, each individual case of notable disease including malaria, must be reported to the Chinese Centre for Disease Control and Prevention (CCDC) through the online Infection Diseases Monitor Information System. The obtained data of all reported malaria cases in Guangdong during the period from 1 January, 2005 to 31 December, 2013 was obtained. The weekly number of cases was aggregated in this study. Demographic data of Guangdong used in this study were collected from the Sixth China Population Census in 2010.

The China Meteorological Data Sharing Service System provided daily data of meteorological measures collected at 36 weather stations in Guangdong from 2005 to 2013. Weekly average/minimum/maximum temperature (°C), cumulative duration of sunshine (hours/week), cumulative precipitation (mm/week), average/maximum/extreme wind speed (m/s), average/minimum/maximum atmospheric pressure (hPa), and average relative humidity (%) were calculated from all of the stations as meteorological measures for the whole territory of Guangdong.

### Statistical analyses

The Granger causality Wald test was performed to examine whether climatic factors could predict malaria incidence (Additional file 1). This method was applied to investigate whether one time-series could predict another, and this association would be invalid when the two series were reversed [22,23]. Statistically significant factors were selected for the following correlation analyses. Spearman correlation analyses were performed to examine the pair-wise correlation among different meteorological factors. To avoid multicollinearity, the highly correlated meteorological factors (r_s_ > 0.7) were not included in the regression model simultaneously [7]. In the final model, weekly average temperature, duration of sunshine and precipitation were considered simultaneously. They may provide different mechanism for malaria transmission and be controlled for while assessing the effects of the other factor.

DLNM was applied to estimate the effects of weekly climatic factors on the weekly number of malaria cases. DLNM is a modelling framework that flexibly describes the non-linear and lagged effects of exposures, and it was originally used in time series analysis [22]. This modelling method has been used widely to assess climatic effects on mortality and morbidity [22,24]. DLNM could simultaneously examine the climate effects over lag and meteorological factors. Gasparrini [25] provided a thorough methodological introduction of DLNM. This study aimed to detect the short-term effects of meteorological factors on malaria cases. Therefore, it is important to control for seasonality and the long-term trend of malaria. Because the weekly number of malaria cases shows an apparent 1-year period (i.e., 52 weeks), the Fourier transformation was used to control for seasonality, as used by Kim *et al*. [7]. The analysis showed that this method produced minimum Q-AIC and had fewer parameters to be estimated compared with the spline method used in a previous study [26]. The long-term trend was controlled for by the inclusion of a linear term of *year* (year = 2005, 2006 … 2013) [7,26]. To check whether the malaria incidence has a non-linear trend across years, a quadratic term of *year* was added into the model and found that the goodness-of-fit of the model was not improved significantly, suggesting that a linear function of year is enough to demonstrate the long-term trend.

The model can be specified as follows:

1 $$ \begin{array}{c}\hfill \log \left(E\left({Y}_t\right)\right)\kern0.5em =\kern0.5em \alpha \kern0.5em +\kern0.5em {\displaystyle \sum_{l=0}^{10}\kern0.3em f\left({T}_{\left(t-l\right)};{\alpha}_l\right)\kern0.5em +\kern0.5em }{\displaystyle \sum_{l=0}^{10}\kern0.3em f\left({S}_{\left(t-l\right)};{\gamma}_l\right)\kern0.5em +\kern0.5em {\displaystyle \sum_{\mathrm{l}=0}^{15}\kern0.3em f\left({P}_{\left(t-l\right)};{\delta}_l\right)}}\hfill \\ {}\hfill \kern3.5em +\kern0.5em \mathrm{ns}\left(\mathrm{mean}\;\mathrm{wind},\ 3\right)\kern0.5em +\kern0.5em  \sin \kern0.5em \left(\frac{2\pi t}{52}\right)\kern0.5em +\kern0.5em  \cos \left(\frac{2\pi t}{52}\right)\kern0.5em +\kern0.5em \mathrm{year}\hfill \end{array} $$

where α is the intercept. *Y*_*t*_ is the number of malaria cases in the week t (*t* = 1, 2, …,471), assumed to follow the quasi-Poisson distribution. *T*_*t-l*_, *S*_*t-l*_ and *P*_*t-l*_ indicate weekly average temperature, duration of sunshine and precipitation with their corresponding coefficients of *α*_*l*_, *γ*_*l*_ and *δ*_*l*_, respectively. Function *f* represents the two-dimensional space of cross-basis functions of natural cubic splines with possible lags. The ranges of lag 0–10, 0–10 and 0–15 weeks were taken to adequately detect the effects of temperature, sunshine and rainfall, respectively [4,6,7]. Natural cubic splines function with degrees of freedom (*df*) 3–5 was used for climatic variables and their lags to determine the overall climate effects (Additional file 2). In the final model, the *df* was specified to be 3 according to the minimum value of Akaike’s Information Criterion for quasi-Poisson (Q-AIC) and the simplicity of the model [27,28]. Relative risk (RR) of malaria morbidity was calculated with reference to the median value for temperature and zero for duration of sunshine and precipitation [4]. Sensitivity analyses were performed by varying *df* (3–5) for lags.

Using the same model, stratified analyses were conducted to identify subpopulation’s susceptibility to meteorological effects by malaria type, gender, and age group. All data analyses were performed in R 3.0.2.

## Results

During the study period of 2005–2013, 1,075 malaria cases were reported in Guangdong with an annual incidence rate of 1.1 cases per 1,000,000 people (Table 1). Most cases were caused by *Plasmodium vivax* (44.9%) and *Plasmodium falciparum* (30.4%) with annual incidence rates of 0.5 and 0.3 per 1,000,000, respectively. Some 856 cases (79.6%) were confirmed by a laboratory test for malaria, and 219 cases (20.4%) were clinically diagnosed (Table 1). The annual incidence rate of men was approximately 3.6 times as high as that of women, and adults suffered from a particularly high risk of malaria compared with the young (0–14 years old).

**Table 1:** **Demographic characteristic of malaria cases in Guangdong, 2005-2013**

**Variables**	**Malaria cases (%)**	**Malaria annual incidence (per 1 million persons)**
**Type of diagnosis**		
Clinical diagnosis*	219 (20.4)	-
Laboratory diagnosis^#^	856 (79.6)	-
**Type of malaria**		
*P. falciparum* malaria	327 (30.4)	0.3
*P. vivax* malaria	483 (44.9)	0.5
Others	265 (24.7)	0.3
**Gender**		
Male	862 (80.2)	1.8
Female	213 (19.8)	0.5
**Age group**		
Young (0–14 years)	35 (3.3)	0.2
Middle (15–59 years)	964 (89.7)	1.3
Old (> = 60 years)	76 (7.1)	1.2
**Total**	1075 (100.0)	1.1

*Diagnosed according to clinical characteristics of patients, including fever, epidemics, periodic chills, coma and convulsions, etc.

^#^Diagnosed by checking P*lasmodium* on the blood smear of patients.

**Figure 2:**
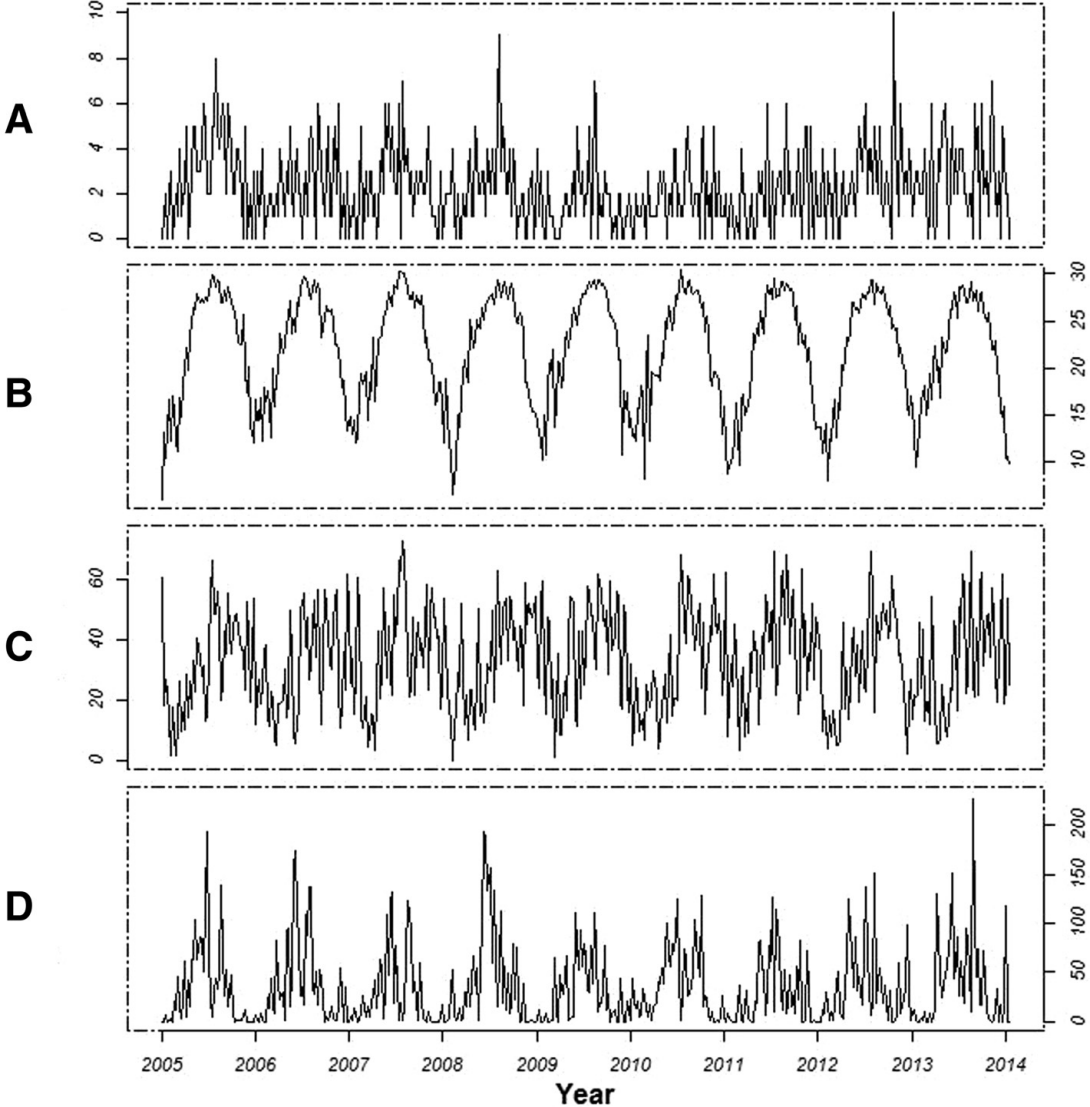
**Time series plots for weekly malaria cases and meteorological factors in Guangdong, China, 2005–2013. A, B, C**, and **D** represent the time series plots of malaria cases, mean temperature, duration of sunshine, and precipitation, respectively.

The obvious one-year seasonal cycle was observed for malaria cases and all meteorological measures (Figure 2). There was a peak in malaria cases in summer and a trough in winter, corresponding to the peaks and troughs in temperature. The duration of sunshine increased dramatically from July to October, while precipitation was higher from April to September.

The Spearman correlation coefficients between the weekly number of malaria cases and mean temperature, duration of sunshine, and precipitation were 0.35 (P < 0.001), 0.14 (P < 0.001) and 0.19 (P = 0.003), respectively.

**Figure 3:**
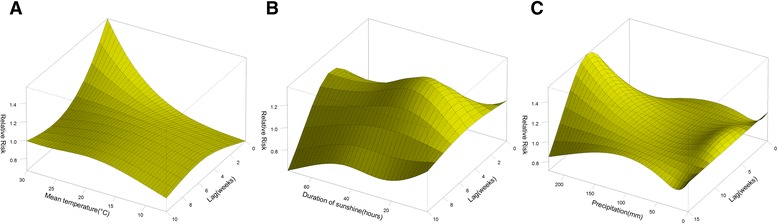
**Relative risks of malaria incidence by climatic factors and lags of weeks. A, B**, and **C** show three-dimensional exposure-response surfaces of temperature, duration of sunshine and precipitation effects, respectively.

**Figure 4:**
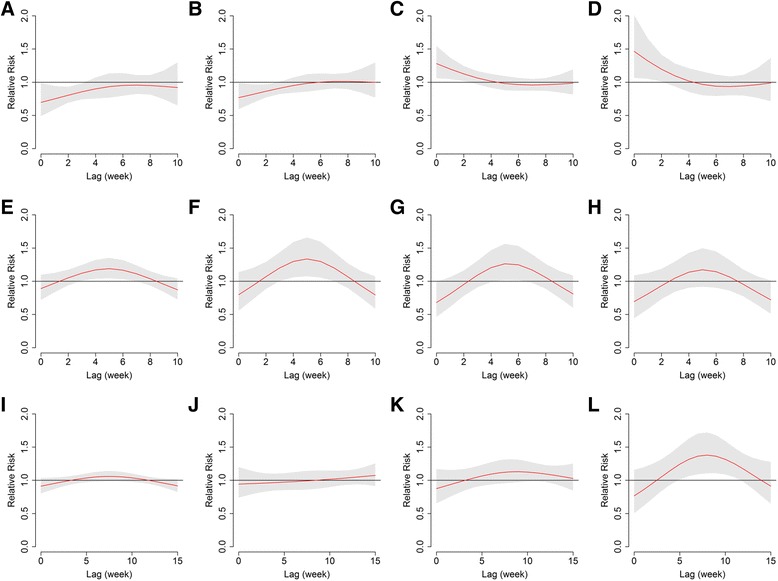
**The estimated lag-response curve at specific meteorology.** The red line represents the estimated relative risks of malaria with shaded bands of 95% confidence intervals. **A, B, C,** and **D** show malaria risk by comparing the fifth (11.8°C), 25th (17.1°C), 75th (27.3°C), and 95th (29.1°C) percentiles of weekly average temperature to the median temperature (23.5°C), respectively. **E, F, G**, and **H** show malaria risk by comparing 10, 20, 40, and 60 hours to 0 hour of weekly sunshine duration, respectively. **I, J, K**, and **L** show malaria risk by comparing 10, 100, 150, and 200 mm to 0 mm of weekly precipitation, respectively.

The three-dimensional exposure-response surfaces reveal the non-linear relationships between these three climatic factors and malaria risks (Figure 3). There were inconsistent patterns across lags. The effects of high temperatures (above the median of 23.5°C) on malaria were positive and significant within the first two weeks (lag 0–2) and decreased gradually with a duration of approximately four weeks (Figures 3A and 4C and D). There was a maximum relative risk (RR) of 1.57 (95% confidence interval (CI): 1.06-2.33) by comparing 30°C to the median temperature at lag 0. The cumulative RR of malaria over lags zero to four were 1.87 (95% confidence interval (CI): 1.12-3.11) and 2.58 (95% CI: 1.10-6.05) by comparing the 75th and 95th to the 50th percentiles of weekly temperature, respectively (Table 2). The effects of low temperature (below 23.5°C) were relatively small but lasted for a period of ten weeks (Figures 3A and 4A and B). The fifth and 25th percentiles of temperature were associated with an increase in malaria risk over lags zero to ten, when compared with the median temperature (Table 2).

**Table 2:** **The effects of meteorological factors on malaria cases along the lag weeks**

	**Relative risk (95% confidence interval)**					
	**Lag 0**	**Lag 4**	**Lag 8**	**Lag 0-4** ^**#**^	**Lag 0-8** ^**#**^	**Lag 0-10/0-15** ^**#**^
**Mean temperature** (°C) (reference value: median)						
11.8	0.70(0.49,0.99)*	0.90(0.76,1.07)	0.95(0.82,1.11)	0.32(0.15,0.70)*	0.26(0.10,0.72)*	0.23(0.06,0.80)*
17.1	0.77(0.59,0.99)*	0.95(0.84,1.07)	1.01(0.90,1.14)	0.47(0.26,0.85)*	0.47(0.21,1.05)	0.48(0.17,1.36)
27.3	1.28(1.06,1.55)*	1.02(0.92,1.13)	0.96(0.87,1.06)	1.87(1.12,3.11)*	1.63(0.82,3.24)	1.57(0.70,3.50)
29.1	1.47(1.07,2.01)*	1.02(0.86,1.21)	0.94(0.80,1.11)	2.58(1.10,6.05)*	2.07(0.67,6.39)	1.98(0.54,7.23)
**Duration of sunshine** (hours/week) (reference value: 0)						
10	0.89(0.72,1.10)	1.17(1.04,1.32)*	1.04(0.94,1.15)	1.20(0.68,2.10)	1.92(0.88,4.21)	1.60(0.66,3.85)
20	0.80(0.56,1.14)	1.30(1.06,1.59)*	1.07(0.90,1.27)	1.24(0.47,3.23)	2.75(0.73,10.38)	2.02(0.45,9.04)
40	0.68(0.47,0.99)*	1.21(0.99,1.47)	1.06(0.89,1.26)	0.69(0.25,1.92)	1.35(0.34,5.42)	1.02(0.21,5.01)
60	0.69(0.44,1.09)	1.14(0.90,1.43)	0.95(0.77,1.17)	0.63(0.18, 2.16)	0.85(0.17,4.40)	0.51(0.08,3.29)
**Precipitation** (mm/week) (reference value: 0)						
10	0.91(0.80,1.03)	1.02(0.96,1.08)	1.06(0.98,1.14)	0.83(0.56,1.25)	1.02(0.60,1.72)	0.94(0.49,1.81)
100	0.94(0.74,1.20)	0.97(0.85,1.10)	1.00(0.87,1.15)	0.79(0.34,1.82)	0.74(0.23,2.35)	0.97(0.21,4.39)
150	0.88(0.66,1.17)	1.03(0.89,1.20)	1.13(0.96,1.32)	0.78(0.30,2.09)	1.15(0.31,4.26)	2.06(0.39,10.81)
200	0.77(0.51,1.16)	1.15(0.94,1.41)	1.38(1.11,1.72)*	0.76(0.19,2.96)	2.33(0.39,13.95)	6.00(0.61,58.96)

^#^The cumulative RRs within lag period.

*Relative risks are statistically significant. 11.8, 17.1, 27.3, and 29.1°C correspond to the 5th, 25th, 75th, and 95th percentile of weekly average temperature, respectively.

A remarkable increase was observed in malaria risk associated with increased weekly duration of sunshine across the range of zero to 20 hours/week, and the risk remained stable when duration of sunshine was above 20 hours/week (Figure 3B). The effects of sunshine were delayed by two weeks, and peaked at lag 5 with a RR of 1.4 (95% CI: 1.1-1.7) when the sunshine duration was 24 hours/week. Figure 4(E-H) shows an inverse U-shaped curve of sunshine-associated malaria risk across lags. When weekly sunshine duration was shorter than 40 hours/week, an increase in sunshine hours was associated with a significant increase in malaria incidence in the following three to seven weeks, while the effects were not statistically significant when the duration was over 40 hours/week.

The malaria risk increased with the ascent of weekly precipitation when the precipitation was larger than 100 mm/week. The effects were delayed by four weeks and peaked at lag 8 with a RR of 1.5 (95% CI: 1.2-2.0) when the precipitation was 225 mm/week (Figure 3C). Figure 4K and L show similar non-linear trends of rainfall-related risk across lags for different precipitation over150 mm/week. When weekly precipitation was beyond 150 mm/week, the rise was associated with a significant increase in malaria incidence at the lag of five to 12 weeks, while the effects were not statistically significant below 150 mm/week.

**Figure 5:**
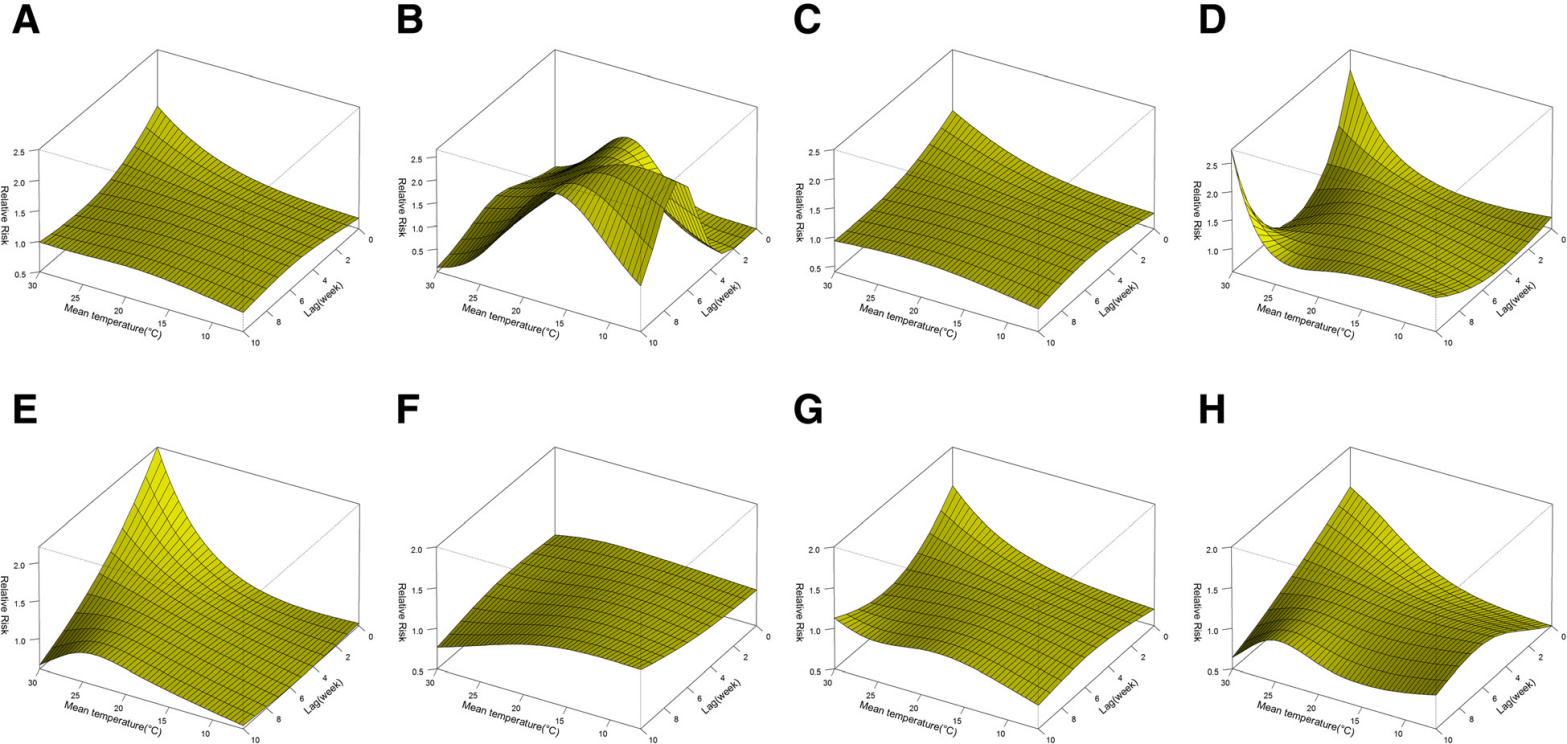
**The relative risks of malaria morbidity by weekly average temperature and lags for subgroups. A, B, C, D, E, F, G**, and **H** show three-dimensional exposure-response surfaces of temperature for the whole population, young, middle-aged, the elderly, *Plasmodium falciparum* malaria, *Plasmodium vivax* malaria, males, and females, respectively. The reference vale was median temperature (23.5°C).

**Figure 6:**
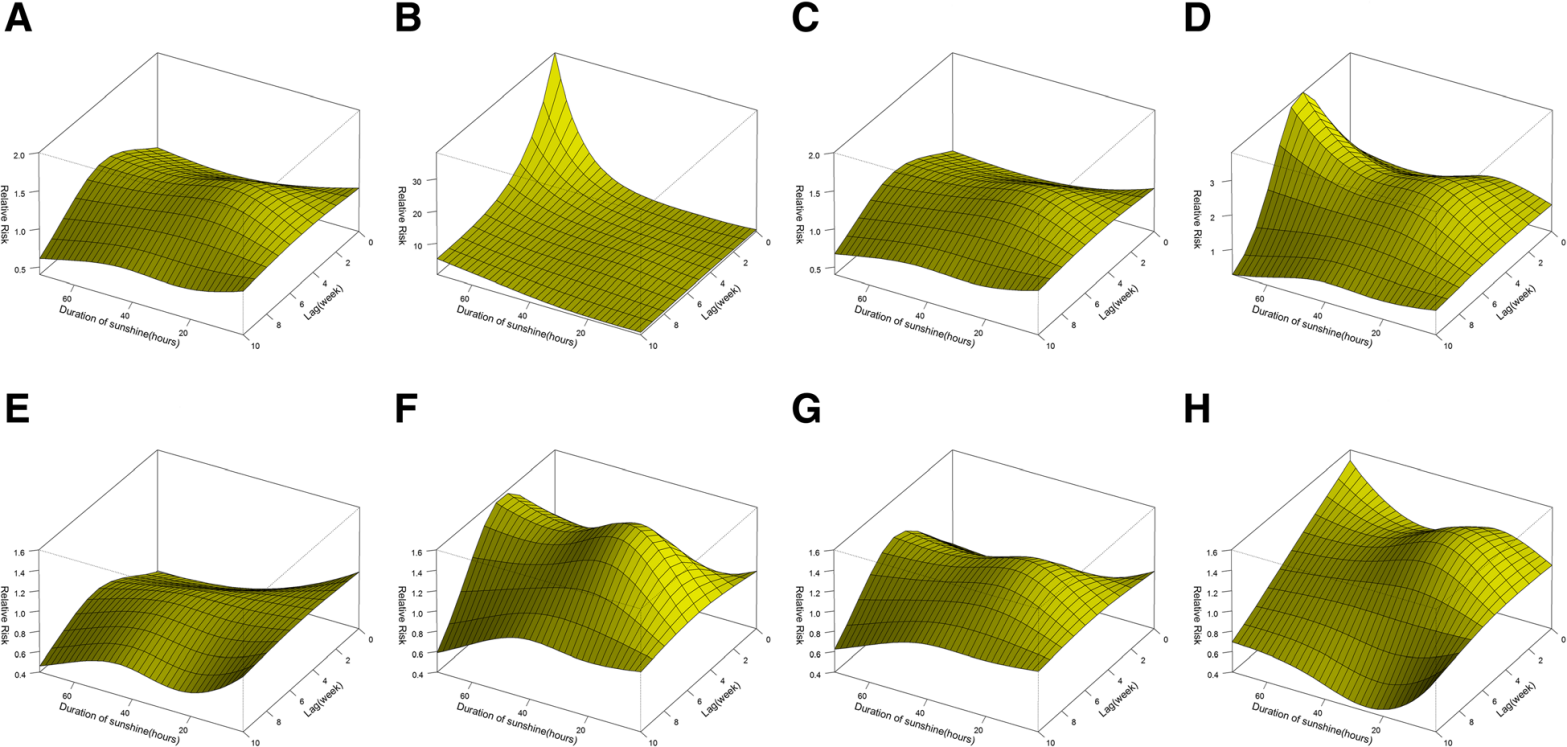
**The relative risks of malaria by weekly duration of sunshine and lags for subgroups. A, B, C, D, E, F, G**, and **H** show three-dimensional exposure-response surfaces of duration of sunshine for the whole population, young, middle-aged, the elderly, *Plasmodium falciparum* malaria, *Plasmodium vivax* malaria, males, and females, respectively. The reference value was 0 hour.

**Figure 7:**
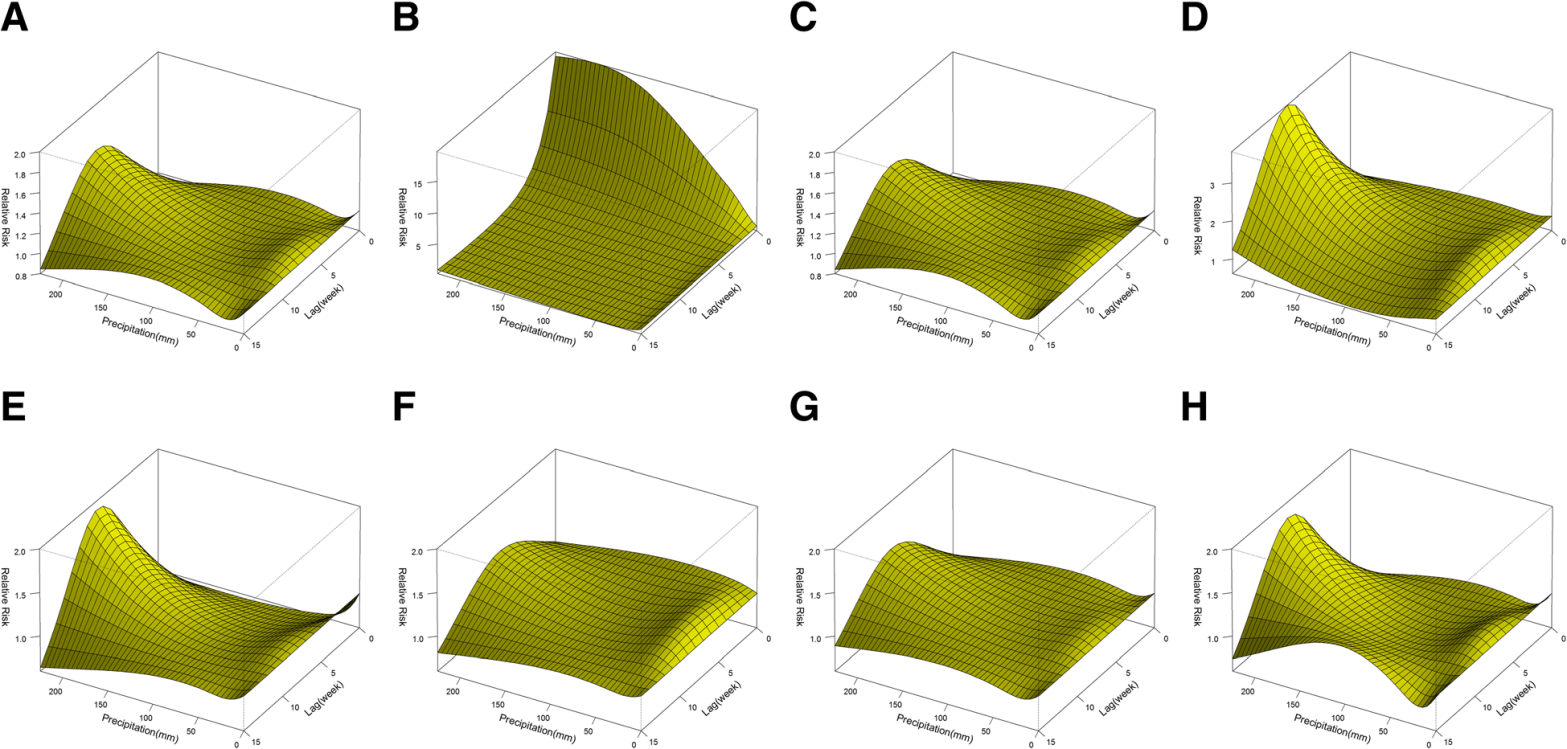
**The relative risks of malaria by weekly precipitation and lags for subgroups. A, B, C, D, E, F, G**, and **H** show three-dimensional exposure-response surfaces of precipitation for the whole population, young, middle-aged, the elderly, *Plasmodium falciparum* malaria, *Plasmodium vivax* malaria, males, and females, respectively. The reference value was 0 mm.

The stratified analyses were conducted by malaria type, gender, and age group. The three-dimensional exposure-response surfaces (Figures 5, 6 and 7) illustrate discrepancies in meteorological effects among different malaria sub-types and sub-groups. The number of *P. falciparum* malaria was affected more by the variations in temperature and precipitation compared with *P. vivax* malaria (Figures 5 and 7). Interestingly, sunshine had negative effects on *P. falciparum* malaria and the RR decreased with the increase of sunshine duration (Figure 6). Males were at a similar temperature-related risk of malaria as females (Figure 5), while females were at a higher risk of sunshine- and precipitation-related malaria than males and the RR peaked without lag when the duration of sunshine was longer than 60 hours/week (Figures 6 and 7). The vulnerability of children and the elderly to the effects of temperature, duration of sunshine, and rainfall was consistently observed (Figures 5, 6 and 7).

Sensitivity analyses on the *df* for lag indicated that the final model was appropriate based on the parsimony with smaller Likelihood Akaike information criteria for quasi-Poison (Q-AIC) value and the stability (Additional file 3).

## Discussion

A much higher malaria incidence rate was found in males than in females in Guangdong, and it was confirmed in the literature that children had lower malaria incidence than adults [2,29]. The changing epidemiology of malaria infection with an increasing proportion of adults and male cases was seen in 34 malaria-eliminating countries [30]. This indicates the increasing importance of outdoor occupational and behavioural factors that put adults, particularly male adults, at much higher chance of endemic exposure to malaria infection compared with children and women. Elderly individuals had similar incidence as the middle-aged people, probably because they had less opportunity for endemic exposure but poorer physiological functions. *Plasmodium vivax* malaria was likely to relapse, which may have a potential influence on the estimate of climatic effects. However, the World Health Organization [31] noted that the relapse rate of malaria would be reduced to 0.6% if patients were treated well. In the present study, the authors believed the small relapse rate of malaria in the data (approximately 1.9%) may not substantially bias the results for the estimation of climatic effects.

The Granger causality Wald test revealed that the mean, minimum, and maximum temperature were all predictors of malaria incidence. The mean temperature could provide more easily interpreted results in a policy context and is more familiar to the public. It is widely used as an indicator of temperature in studies of relationships between vector-borne diseases and climate [7,10,22]. However, some studies used minimum temperature, maximum temperature, or both as main predictors, which may promote examining the effects of extreme temperature, but there were strong correlations between these temperature measures, and the simultaneous inclusion of these measures in the model may complicate the model with higher Q-AIC value and invalidate the parameter estimation [7,10,32]. In the present study, weekly average temperature as the only temperature parameter was included in the model to assess the temperature effect on malaria incidence. The positive and non-linear relationship was found between mean temperature and malaria incidence. The immediate effects of high temperature (above 23.5°C) had a duration of four weeks, while low temperature (below 23.5°C) was associated with an increase in malaria risk within the following zero to ten weeks. The biological mechanism for the different time responses to high and low temperature remains unclear. Similarly, many studies have showed that the effects of low temperature on human mortality persisted for much longer than that of high temperature [33,34]. It is possible that high temperature (over the median temperature) mainly affects mosquito feeding habits and activity behaviours and the development of parasites in mosquitoes within a short time, while low temperature inhibits the survival of mosquitoes and fundamentally reduces the possibility of malaria infection for a longer period. Rueda *et al*. [35] noted that high temperature could decrease the body size, increase head capsule width, lengthen the larval body, add weight to the instars or pupae of mosquitoes, and increase the survival from eclosion to adult. Increasing temperature within proper scope can speed up the spreading of malaria vectors, shorten the growth period of parasites and cause fast transmission and high malaria incidence [15]. However, it is necessary to be cautious to explain and generalize the lag 0 effect of temperature. It is speculated that climate factors may have indirect effects on malaria incidence during the incubation period. It is noted that high temperature could accelerate the activities of mosquitoes and increase the bites on human [30], which may increase the rate of malaria infection at the current week. Further experimental studies on the underlying biological mechanism would be helpful to determine the time-course of the effects of ambient temperature on malaria.

Therefore, the effects of high temperature were immediate, but effects of low temperature were delayed. The evidence suggests that some measures are needed to mitigate the effects in warm seasons, such as installing air conditioners, reducing outdoor activities, and conducting some anti-mosquito operations.

The literature showed discrepancies in the lag period of temperature effect. Several previous studies showed there was little effect of temperature after ten weeks [4,6,7], while Craig and Briët *et al*. [5,12] reported a lag of nine to 16 weeks, which may be caused by the specific single lags chosen and the broader time scale (e.g., monthly) used. Furthermore, the regional differences in the proportions of malaria sub-types may also contribute to these discrepancies. Additionally, some previous findings were based on simple models, including only one or two climatic factors. In the present study, the effects were evaluated after adequately controlling for seasonality, trend, and potential confounding of other meteorological factors. Further multiple-region analyses using unified modeling strategies are required to better understand the lagged effects of temperature to identify the optimum time scale of supervision and effective control.

No temperature threshold was observed for the effect on malaria in Guangdong. Similarly, Wardrop and Bi *et al.* [10,32] did not find a temperature threshold for *P. vivax* malaria in two Chinese subtropical cities. However, Kim *et al*. [7] observed a temperature threshold of −4°C that was an optimum temperature, above or below which the risk of malaria increased, whereas the malaria risks changed slightly above 24°C in Korea. This is possibly because the relatively shorter range of temperatures in sub-tropical regions and relatively smaller temperature gradient could not provide the full picture of the exposure-response relationship. In Guangdong, the range of weekly average temperature during the study period was 6.0 to 30.5°C compared with −8.5 to 27.5°C in the Korea study [7]. Additionally, the effects of hot temperature on *Plasmodium falciparum* malaria were greater than on *P. vivax* malaria. *P. falciparum* goes through the whole life cycle within a shorter time at 28-30°C [36]. Hot temperature favours *P. falciparum* development and may have a significant influence on mosquito infection rates and parasite densities [37]. *Plasmodium vivax* could complete growth and development within a broader range of 15 to 30°C and therefore may be less sensitive to temperature changes than *P. falciparum*.

Previously, only Kim *et al*. [7] considered the delayed effects of sunshine. In the present study, a statistically significant negative effect of sunshine duration on malaria was found at the same week, while the effects were positive at lag three to seven weeks. Furthermore, falciparum malaria and vivax malaria showed differences in the dose–response relationship with sunshine. Li [38] also found that there were positive associations between the monthly duration of sunshine and malaria cases in the current month. Zhang *et al.* and Ayala *et al*. [39,40] did not find a significant relationship between sunshine and concurrent malaria cases. On the contrary, a negative monotonic impact of the duration of sunshine on malaria incidence was observed in Kim’s study in Korea, where only *P. vivax* malaria was considered [7]. This discrepancy may have been caused by the differences in the types of main vectors for malaria between China and Korea. The predominant vectors for *P. vivax* are *Anopheles lesteri* and *Anopheles sinensis* in Korea [41]. The former has higher sensitivity to malaria and proved to be heliophobic [42]. However, the main vector for *P. vivax* is *An. sinensis* in mainland China [43]. It is evident that the development of *An. sinensis* is positively associated with the duration of sunshine [44]. While the main vectors for *P. falciparum* are *Anopheles minimus* and *Anopheles dirus*, which are usually active in the shadows [45,46]. There is a need to further explore the effects of sunshine on specific vectors and malaria subtypes.

Precipitation was the most controversial factor in previous studies of climatic effects on malaria incidence. A study found significant rainfall effects when precipitation was 2.4 times higher than the normal level [47]. Rainfall plays an important role in the survival of mosquitoes, but it may contribute little to the saturation if there has been sufficient rainfall or the sunshine effects are considered, because a small amount of water could evaporate quickly in strong sunlight [4,7]. Wardrop *et al*. [10] elucidated there was no significant effect when rainfall was less than 100 mm/month in Yunnan, China. A study in Ethiopia found that rainfall had an important influence on malaria incidence in hot districts with an altitude lower than 1,650 m, but not in cold districts with an altitude higher than 1,650 m [6]. In the present study, there were significant effects of weekly rainfall over 150 mm/week, approximately 4.4 times as much as the mean precipitation of 34.2 mm/week. This is possibly because most areas in Guangdong have advanced drainage systems so that low levels of precipitation do not facilitate the development of mosquito larval habitats or mosquito transmission and therefore have no significant effect on malaria incidence. The positive effects of precipitation observed in this study had a lag of four weeks and peaked at lag of eight weeks, similar to previous findings [6,7]. The lagged effects were reasonable because it takes time to gather rainfall into little pools and for mosquito to lay eggs on them, consequently affecting mosquito density and malaria incidence. Consistent with Yan *et al*. [32], the present study indicated that precipitation-related effects for *P. falciparum* and *P. vivax* were similar, but the effects were relatively higher for *P. falciparum* than the other. This is probably due to the biological difference in the parasite adaptation, mosquito habits, and the climatic sensitivity between these two types of Plasmodium.

To our knowledge, no study has investigated the potential effect of gender and age on meteorology-associated risks of malaria. In the present study, no gender difference in the sensitivity to temperature-associated malaria risk was found, but females were at a higher risk of sunshine- and precipitation-related malaria. Further, it was found that the young and the elderly were more susceptible to temperature-, sunshine- and precipitation-associated malaria. Their vulnerable immune system, and higher likelihood of experiencing mosquito bites, may contribute to children’s susceptibility. Loss of physical function and poorer general health may put the elderly at high risk of weather-related malaria. This is supported by several studies that noted the special vulnerability of children and old people to climate change on public health [48,49].

There are several limitations of the present study. Firstly, although there is a long-standing comprehensive surveillance system of malaria in Guangdong and each case must be reported to the national CCDC, the under-reporting of malaria cases is inevitable. Secondly, the effects of extreme weather events, such as heat waves and floods, were not considered, which may play an important role in malaria pandemics. Although the nine-year data were utilized as much as possible, the analyses on a weekly basis were performed and significant effects of some metrological factors may not have been detected because of the limited number of cases, particularly for some specific subgroups using stratified analyses. Further analyses based on a longer study period or daily data if appropriate may promote a better understanding of climatic effects.

## Conclusions

Malaria remains an important public health problem in Guangdong, China. Malaria cases cluster in male adults. It was found that mean temperature, followed by weekly duration of sunshine and weekly precipitation, had delayed and non-linear effects on malaria incidence, particularly for susceptible sub-populations. The findings promote a better understanding of climatic effects on malaria and provide important information for malaria prediction. Further combinations with data from past decades or other regions and the consideration of extreme weather events could be used to construct a malaria warning system.

## Additional files

Additional file 1:
**The results of the Granger causality Wald tests for climate factors.**
Additional file 2:
**Likelihood Akaike’s information criteria for quasi-Poison (Q-AIC) values for different models.** All the dfs were selected from 3–5 for mean temperature, precipitation, duration of sunshine and mean wind with smallest Q-AIC value. The bold value refers to the Q-AIC value of the final model. Additional file 3:
**Sensitive analyses with Likelihood Akaike’s information criteria for quasi-Poison (Q-AIC) values for different models.** The Q-AIC values of models with different degrees of freedom for lags. The bold value refers to the Q-AIC value of the final model.
