# Supplementary material for: Malaria incidence from 2005–2013 and its associations with meteorological factors in Guangdong, China
Source: Malar J. 2015 Mar 18;14:116. doi: 10.1186/s12936-015-0630-6 (PMC4389306; doi:10.1186/s12936-015-0630-6)
Supplement: Additional file 1: — The results of the Granger causality Wald tests for climate factors. [file 12936_2015_630_MOESM1_ESM.pdf]

Additional file 1: The results of the Granger causality Wald test for climate factors:

Climate factors	Chi-square	Degree of freedom	P-value
Mean temperature (^oC)	38.85	15	<0.001
Minimum temperature (^oC)	34.42	15	0.003
Maximum temperature (^oC)	37.59	15	0.001
Precipitation (mm/week)	37.53	15	0.001
Duration of sunshine (hour/week)	29.79	15	0.013
Mean wind speed (m/s)	32.90	15	0.005
Mean relative humidity (%)	32.33	15	0.006
Minimum atmosphere pressure (hPa)	29.84	15	0.013
Maximum atmosphere pressure (hPa)	44.43	15	<0.001
Mean atmosphere pressure (hPa)	36.11	15	0.002
Diurnal temperature range (^oC)	21.23	15	0.130
Maximum wind speed (m/s)	11.83	15	0.692
Extreme wind speed (m/s)	12.00	15	0.679
