# Supplementary material for: Malaria incidence from 2005–2013 and its associations with meteorological factors in Guangdong, China
Source: Malar J. 2015 Mar 18;14:116. doi: 10.1186/s12936-015-0630-6 (PMC4389306; doi:10.1186/s12936-015-0630-6)
Supplement: Additional file 2: — Likelihood Akaike’s information criteria for quasi-Poison (Q-AIC) values for different models. All the dfs were selected from 3–5 for mean temperature, precipitation, duration of sunshine and mean wind with smallest Q-AIC value. The bold value refers to the Q-AIC value of the final model. [file 12936_2015_630_MOESM2_ESM.pdf]

Additional file 2: Likelihood Akaike information criteria for quasi-Poison (Q-AIC) values for different models:

All the dfs were selected from 3-5 for mean temperature, precipitation, duration of sunshine and mean wind with smallest AIC. The bold value refers to the Q-AIC value of the final model.

df of wind speed	df of duration of sun	df of temperature	df of precipitation		
			**3**	**4**	**5**
3	3	3	**1697.5**	1701.5	1701.5
3	3	4	1702.8	1706.6	1706.9
3	3	5	1709.1	1713.5	1713.9
3	4	3	1705.0	1708.1	1707.9
3	4	4	1710.1	1715.7	1713.1
3	4	5	1716.6	1719.8	1720.3
3	5	3	1708.0	1710.1	1710.5
3	5	4	1713.2	1714.6	1715.5
3	5	5	1718.7	1720.7	1721.2
4	3	3	1699.7	1703.6	1703.3
4	3	4	1705.0	1708.8	1708.9
4	3	5	1711.3	1715.6	1715.7
4	4	3	1707.1	1710.2	1709.8
4	4	4	1715.4	1714.9	1715.1
4	4	5	1718.7	1721.9	1722.1
4	5	3	1710.3	1715.3	1715.5
4	5	4	1715.6	1717.0	1717.6
4	5	5	1720.9	1722.9	1723.2
5	3	3	1701.3	1705.3	1705.3
5	3	4	1706.7	1710.5	1710.9
5	3	5	1715.9	1717.4	1717.8
5	4	3	1708.9	1715.1	1711.8
5	4	4	1714.1	1716.8	1717.2
5	4	5	1720.5	1723.9	1724.2
5	5	3	1715.4	1714.6	1714.9
5	5	4	1717.7	1719.3	1720.0
5	5	5	1723.0	1725.3	1725.6
