# Supplementary material for: Malaria incidence from 2005–2013 and its associations with meteorological factors in Guangdong, China
Source: Malar J. 2015 Mar 18;14:116. doi: 10.1186/s12936-015-0630-6 (PMC4389306; doi:10.1186/s12936-015-0630-6)
Supplement: Additional file 3: — Sensitive analyses with Likelihood Akaike’s information criteria for quasi-Poison (Q-AIC) values for different models. The Q-AIC values of models with different degrees of freedom for lags. The bold value refers to the Q-AIC value of the final model. [file 12936_2015_630_MOESM3_ESM.pdf]

Additional file 3 Sensitive analyses with Likelihood Akaike information criteria for quasi-Poison (Q-AIC) values for different models:

The Q-AIC values of models with different degrees of freedom for lags. The bold value refers to the Q-AIC value of the final model.

df for lags	Q-AIC
3	**1697.5**
4	1713.5
5	1715.9
